# Malaria outbreak in Riaba district, Bioko Island: lessons learned

**DOI:** 10.1186/s12936-020-03347-w

**Published:** 2020-08-03

**Authors:** Carlos A. Guerra, Godwin Fuseini, Olivier Tresor Donfack, Jordan M. Smith, Teresa Ayingono Ondo Mifumu, Gninoussa Akadiri, Delicias Esono Mba Eyang, Consuelo Oki Eburi, Liberato Motobe Vaz, Victor Mba Micha, Leonor Ada Okenve, Christopher R. Janes, Ramona Mba Andeme, Matilde Riloha Rivas, Wonder P. Phiri, Michel A. Slotman, David L. Smith, Guillermo A. García

**Affiliations:** 1 Medical Care Development International, 8401 Colesville Road, Suite 425, Silver Spring, MD 20910 USA; 2 MedicalCare Development International, Av. Parques de Africa, Malabo, Equatorial Guinea; 3 National Malaria Control Programme, Ministry of Health and Social Welfare, Malabo, Equatorial Guinea; 4 Department of Entomology, Texas A&M University, TAMU 2475, College Station, TX 77843 USA; 5 Institute for Health Metrics and Evaluation, University of Washington, 2301 Fifth Avenue, Seattle, WA 98121 USA

## Abstract

At the beginning of 2019, a sudden surge of malaria cases was observed in the district of Riaba, Bioko Island. Between January and April, confirmed malaria cases increased 3.8-fold compared to the same period in 2018. Concurrently, anopheline human biting rate (HBR) increased 2.1-fold. During the outbreak, 82.2% of the district population was tested for malaria with a rapid diagnostic test; 37.2% of those tested had a detectable infection and were treated according to national guidelines. Vector control interventions, including indoor residual spraying and larval source management were scaled-up. After the interventions, the number of confirmed cases decreased by 70% and the overall parasite prevalence in the communities by 43.8%. Observed prevalence in a follow up malaria indicator survey, however, was significantly higher than elsewhere on the island, and higher than in previous years. There was no significant reduction in HBR, which remained high for the rest of the year. The surge was attributed to various factors, chiefly increased rainfall and a large number of anthropogenic anopheline breeding sites created by construction works. This case study highlights the need for sustained vector control interventions and multi-sector participation, particularly in malaria control and elimination settings with persistently high local malaria receptivity.

## Background

**Fig. 1:**
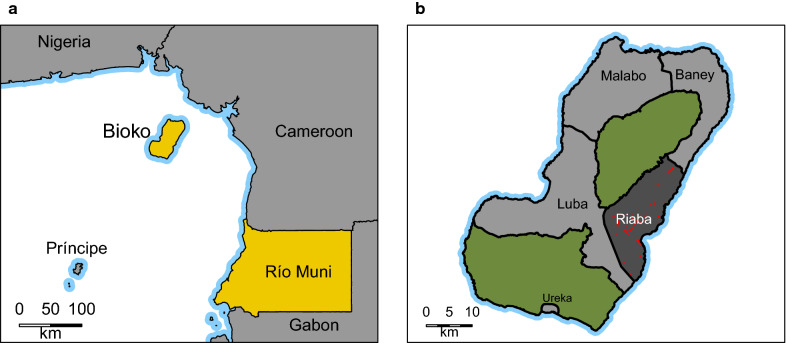
Bioko Island and Riaba district. **a** Location of Bioko Island in the Gulf of Guinea, off the coast of Cameroon and to the Northwest of the continental region of Equatorial Guinea (Río Muni). **b** Administrative divisions of Bioko showing Riaba district in darker grey. The red points indicate communities within the district. The green areas are nature reserves. Ureka is part of Luba district

Bioko is the largest island of Equatorial Guinea. Administratively, it is divided into four districts: Malabo, Baney, Luba and Riaba (Fig. 1). The island has a population of about 270,000 people, mostly concentrated in Malabo, the main urban centre and country capital. The rural district of Riaba, in the Southeast, has a resident population estimated at 2560 inhabitants (unpublished data from a household and health population census conducted in 2018).

In 2004, the Bioko Island Malaria Control Project (BIMCP; now the Bioko Island Malaria Elimination Project—BIMEP) was established in order to scale-up malaria interventions on the island. The BIMEP periodically distributes long-lasting insecticidal nets (LLINs) through mass-campaigns, implements annual indoor residual spraying (IRS) rounds, conducts annual malaria indicator surveys (MIS) and runs continuous entomological monitoring in sentinel sites across the island. In collaboration with the National Malaria Control Programme (NMCP), the BIMEP provides malaria case management training and supervision as well as anti-malarial drugs and diagnostic tools free of charge in all public health facilities on the island. The project has also supported Bioko’s health information system through the implementation of the District Health Information System 2 platform for health facility data in the public sector.

Historically, malaria on Bioko Island was hyper to holoendemic, with year-round transmission. Malaria interventions implemented by the NMCP/BIMEP have successfully reduced parasite rate (PR) by around 75% [1]. In Riaba district, prevalence in all ages measured in recent years has ranged between a low of 6.8% in 2016 and a high of 12.1% in 2018. Before control, malaria transmission in Riaba district was particularly intense. An entomological survey conducted in that district between 1998 and 1999 estimated an annual entomological inoculation rate (EIR) of 1030 infected bites per person per year (ib/p/y) (242 ib/p/y for *Anopheles gambiae* sensu stricto (*s.s.*) and 788 ib/p/y for *Anopheles funestus*) [2], an estimate that far exceeded all EIR recorded elsewhere across sub-Saharan Africa [3, 4], with one exception from Uganda [5]. In 2009, after 5 years of intensive interventions, annual EIR in one of the entomological monitoring sites in Riaba district (Patio Balboa) was measured at 311 ib/p/y, exclusively due to *An. gambiae s.s.* M form (later renamed *Anopheles coluzzii* [6]) and *Anopheles melas* [7]. No specimens of *An. funestus* and *An. gambiae s.s.* S form were found in this or other recent surveys and are considered eliminated on the island [8–10].

In this case study, a recent malaria outbreak that developed at the beginning of 2019 in Riaba district is described. The response from the NMCP/BIMEP teams as well as the outcome of this response are documented. Finally, the most plausible drivers for this unexpected outbreak as well as some of the major challenges for malaria control and elimination on Bioko Island are discussed.

## The outbreak

**Fig. 2:**
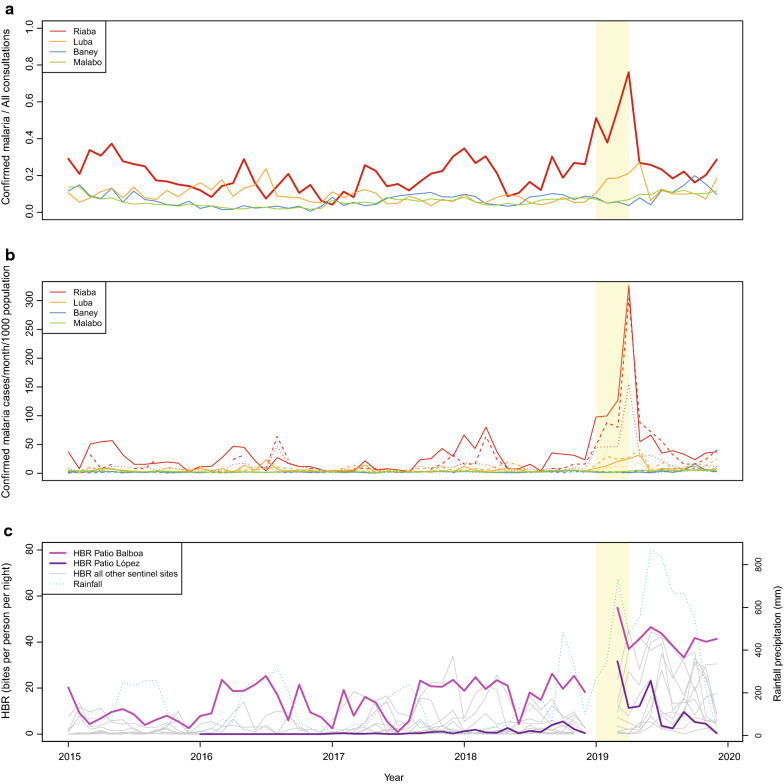
Confirmed malaria cases, malaria incidence and HBR on Bioko Island, by district, 2015–2019. The light-yellow shading highlights the period between January to April 2019. **a** Ratio of confirmed malaria cases to all outpatient consultations. **b** Malaria incidence by district and age-group. Solid, dashed and dotted lines represent the 2–12, 13–17 and 18–50 years old age groups. **c** HBR estimates from the 12 entomological monitoring sentinel sites and rainfall data for the whole of Bioko Island. Entomological monitoring did not take place in January and February 2019, hence the missing data for these months. Rainfall data were digitized from https://www.worldweatheronline.com

In the first third of 2019, records from the Riaba district hospital revealed a 3.8-fold increase in the number of confirmed malaria cases relative to the same period in 2018 (Fig. 2a). Health information system data documented 874 confirmed malaria cases between January and April and a corresponding increase in the ratio of confirmed malaria cases to all outpatient consultations, with mean of 0.55, ranging from 0.38 in February to 0.76 in April. This was significantly higher than this ratio observed in Riaba for the same months in the preceding 4 years (mean 0.19, IQR 0.14–0.26) as well as in the other three districts between 2015 and 2019 (mean 0.06, IQR 0.04–0.08; Fig. 2a). The peak in malaria cases was reflected in an incidence of more than 300 cases per 1000 people in children between 2 and 17 years old in April 2019, significantly higher than any record since 2015. Malaria incidence in adults in that month was also significantly higher, with 155 cases per 1000 people in those aged between 18 and 50 years old (Fig. 2b).

### Entomological monitoring

During concurrent entomological monitoring in the two sentinel sites located in Riaba district, Patio Balboa and Patio López, significant increases in human biting rate (HBR) were observed. HBR was determined from human landing catches that were performed once a month for two consecutive days in four houses at each site, as described elsewhere [7]. Briefly, two volunteers were positioned in each house, one indoors and one outdoors, between 19h00 and 06h00, switching positions at midnight. HBR, therefore, corresponded to indoor and outdoor biting estimates. During March and April 2019, 735 *Anopheles* were captured in Patio Balboa and 344 in Patio López. Due to logistical constraints, no human landing catches were conducted in January and February. The monthly HBR for the same months was determined at 54.9 and 37.0 bites per person per night (b/p/n) in Patio Balboa, and 31.6 and 11.4 b/p/n in Patio López (Fig. 2c). For comparison, the average HBR in these two months for the other ten sentinel sites outside Riaba district was 10.0 b/p/n and Patio Balboa alone contributed 27.5% of the vectors collected in all 12 sites across Bioko.

Importantly, species composition changed in Patio Balboa between 2018 and 2019, but remained relatively stable in Patio López. Amongst 541 specimens collected in Patio Balboa between March and December 2019, 89.6% were *An. coluzzii* and 10.4% were *An. melas*. In 2018, *An. coluzzii* represented 75.3% of the captures. This increase in the proportion of *An. coluzzii* was caused by an increase in HBR for this species, from 18.34 b/p/n in 2018 to 38.7 b/p/n in 2019, together with a slight decrease in *An. melas* HBR from 5.07 b/p/n to 3.13 b/p/n. In Patio López, *An. melas* represented the majority (62.8%) of 299 specimens collected between March and December 2019; 37.2% were identified as *An. coluzzii*, a proportion similar to the one observed in 2018 for this species (35.1%). The HBR for both species increased at this site, from 0.74 b/p/n to 3.96 b/p/n for *An. coluzzii* and from 1.03 b/p/n to 6.13 b/p/n for *An. melas*.

In Patio Balboa, *Plasmodium falciparum* sporozoites were found in six *An. coluzzii* in 2019, representing a sporozoite rate of 1.2%, and no sporozoites were found in *An. melas*. This translated into an estimated EIR of 174.7 ib/p/y in 2019, whereas in 2018 EIR was estimated at 15.6 ib/p/y with a sporozoite rate of 0.8% (unpublished data). In Patio López, no sporozoites were found in the specimens collected in 2019, which was also the case in 2018. *Anopheles* species identification and sporozoite detection were both done on head and thorax using PCR-based analyses [7, 11, 12].

### The response

**Fig. 3:**
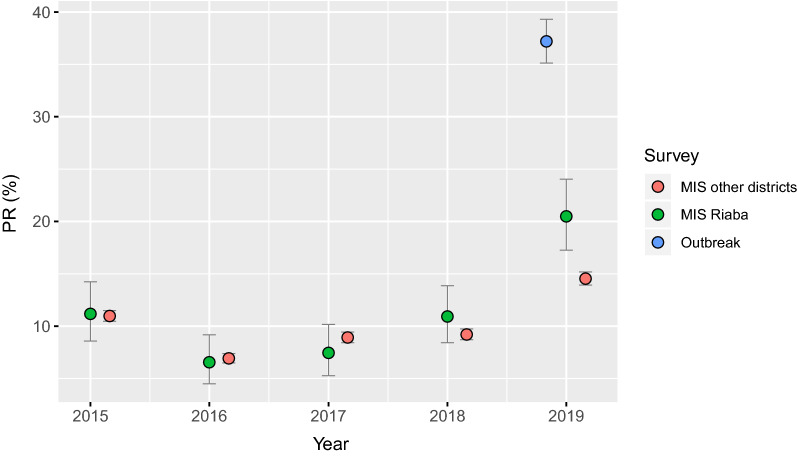
Malaria prevalence estimates from MIS and from the outbreak survey. Data from MIS were plotted separately for Riaba and for the rest of the island. Error bars illustrate the 95% confidence intervals. Individuals with history of off-island travel in the previous 8 weeks were excluded from the MIS data to control for the confounding effects of malaria importation [22]

**Fig. 4:**
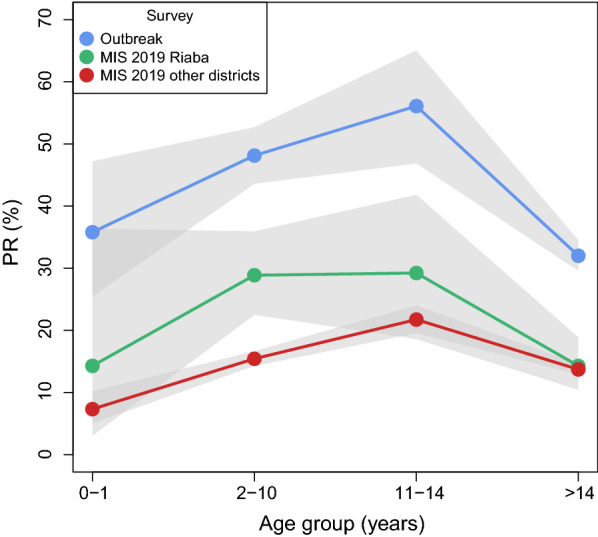
Malaria prevalence by age group. The grey shaded areas illustrate the 95% confidence intervals. Individuals with history of off-island travel in the previous 8 weeks were excluded from the MIS data to control for the confounding effects of malaria importation [22]

In response to the outbreak, the NMCP/BIMEP teams reinforced community sensitization, malaria diagnosis and treatment, and vector control. First, the communications component mobilized the population in Riaba through community leaders to raise malaria prevention awareness and to promote acceptance of case detection, treatment and IRS. Second, the NMCP/BIMEP engaged 13 community health workers to test the population in Riaba for malaria using rapid diagnostic tests (RDTs; CareStart Malaria Pf/PAN (HRP2/pLDH) Ag Combo, ACCES BIO, Inc.). This was done as part of a test and treat strategy whereby the whole district population, including all age groups, was targeted (Table 1). Between 22 and 30 April, 2105 consenting individuals who were present at the time were tested for malaria. In the case of young children, consent was obtained from their guardians. This represented 82.2% of the district population and, though a complete census was attempted, technically constituted a non-probability sample. Amongst those tested, 783 had RDT detectable infections, resulting in an estimated PR of 37.2% (95CI 35.1–39.3%). This was significantly higher than prevalence estimates in Riaba district measured during MIS in the four previous years (Fig. 3). Investigation by age groups revealed that prevalence was higher in children, with 48.1% (95CI 43.6–52.7%) of those between 2 and 10 years old and 56.1% (95CI 46.8–65.0%) of those between 11 and 14 years old found infected (Fig. 4). All individuals who tested positive were treated with artesunate-amodiaquine, according to national treatment guidelines at the time.

**Table 1:** Prevalence surveys conducted in Riaba district in 2019

Survey	Dates	Sample	Age range	Sample size	Positive (% [95CI])
Outbreak	22–30 Apr	All present	All ages	2105	783 (37.2 [35.1–39.3])
MIS	12–18 Aug	Representative	All ages	571	117 (20.5 [17.3–24.0])

The outbreak survey was part of a test and treat strategy. MIS are implemented annually on Bioko since 2004 and, in 2019, this survey served as a follow up to the outbreak survey

**Fig. 5:**
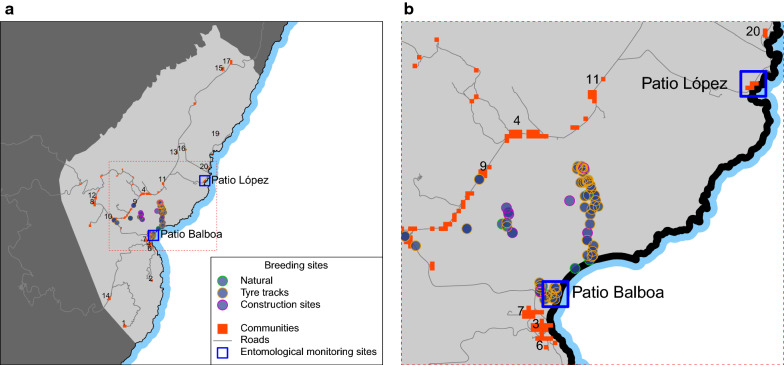
Location of communities and mapped anopheline breeding habitats in Riaba district. **a** Riaba is shaded in lighter grey. Breeding sites are marked according to type as natural or anthropogenic (*i.e.* construction sites and tyre tracks). Patio López and Patio Balboa are highlighted as the two longitudinal entomological monitoring sites in Riaba district. The numbers identify the other communities. **b** Detail of the area around the construction sites (red box in **a**)

**Fig. 6:**
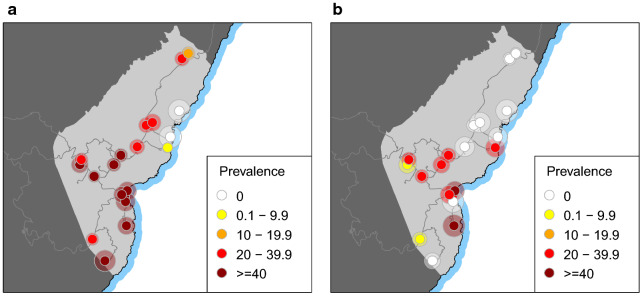
Malaria prevalence estimates by community in Riaba district. Riaba is shaded in lighter grey. **a** Outbreak survey. **b** 2019 MIS. The shaded areas around the mapped points are proportional to the 95% confidence intervals of the prevalence estimates

**Fig. 7:**
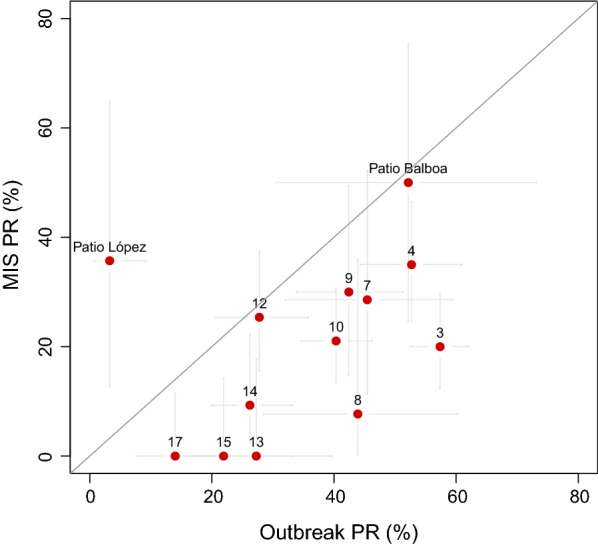
Prevalence estimates in Riaba district during the outbreak against the 2019 MIS. Horizontal and vertical lines illustrate the 95% confidence intervals of the estimates. Sites where less than 10 people were sampled are not shown. Numbers correspond to the communities shown in Fig. 5. Patio Balboa and Patio López are named specifically

Between 22 and 27 April 2019, as part of the annual IRS round, 757 of 871 inhabited households in the district were sprayed with pirimiphos-methyl (Actellic 300CS, Syngenta AG), achieving a coverage of 86.9% and effectively protecting the entire district population. The choice of insecticide was based on a previous investigation of insecticide resistance profiles for *An. gambiae* sensu lato and of the residual activity of pirimiphos-methyl on Bioko. The findings of that study showed that vectors were susceptible to the insecticide and that the residual activity was 8 months [13]. Finally, the entomology team was deployed to search for and treat larval sources across the district. Their main finding was a substantial number of breeding sites created by major construction developments that had established recently (Fig. 5). A total of 1241 breeding sites were mapped during 2019 using global positioning systems enabled mobile devices running ArcGIS Collector (Esri, Inc.). Of these, 1119 (90.2%) were described as anthropogenic: 122 created directly by the construction sites and 997 corresponding to tyre tracks related to these infrastructure development projects. Amongst the mapped anthropogenic breeding sites, 37.8% were found positive for anopheline immature stages upon evaluation. Considerably more potential anthropogenic breeding habitats were found, but mapping, evaluating and treating all of them proved impossible as this effort overwhelmed the manpower capacity available. Notably, communities closer to these larval habitats showed higher malaria prevalence (Figs. 6 and 7).

### The outcome

Following interventions, the incidence of malaria in May 2019 dropped to 54.7 and 88.0 cases per 1000 inhabitants in the 2 to 12 and 13 to 17 years-old age groups, and the mean ratio of malaria consultations to all consultations for the remainder of the year was 0.23 (range 0.16–0.29; Figs. 2a, b). The HBR at Patio Balboa, however, remained high for the rest of the year, with a mean of 40.8 b/p/n (range 33.3–46.5 b/p/n) between May and December 2019. In contrast, mean HBR at Patio López was 7.6 b/p/n (range 0.4–23.1 b/p/n) for the same period (Fig. 2c).

Between 12 and 18 August 2019, during the annual MIS, 571 people of all ages from a representative, random sample of the whole district population were tested with RDTs (Table 1). This survey served as a follow up to the prevalence survey conducted in April 2019, during the outbreak. A total of 117 individuals had RDT detectable infections, representing a PR of 20.5% (95CI 17.3–24.0%; Fig. 3), a significant reduction compared to the outbreak survey. Importantly, however, this estimate proved significantly higher than that for the rest of the island during the same MIS as well as significantly higher than PR estimates from the previous four years, both for Riaba district and for the rest of the island. The age profile of the 2019 MIS PR showed spikes in the 2 to 10 and 11 to 14 years-old age-groups, similar to those observed during the outbreak survey (Fig. 4). Disaggregating the PR by community showed that, despite the overall decrease, this was more evident in the North and South of the district, with a cluster of higher prevalence persisting in communities around the area where the breeding habitats associated with construction sites had been identified (Figs. 6 and 7).

## Conclusions

The sudden increase in malaria morbidity in Riaba district in the first third of 2019 coincided with a significant increase in rainfall patterns and anopheline HBR that were observed across the island (Fig. 2). Even though other areas had seen an increase in confirmed malaria cases, namely in Luba district and in certain isolated communities in the West of Malabo district, in none was the spike in cases as dramatic as the one observed in Riaba district. The response from the malaria control teams to diagnose and treat positive individuals with anti-malarials was certainly effective and successfully curbed malaria cases by May 2019. Malaria prevalence and HBR, however, remained high, despite vector control interventions (Figs. 2 and 3). The significantly higher malaria prevalence in 2 to 10 year-old children measured almost 4 months later during the 2019 MIS (Fig. 4) could be explained by higher parasite densities in children that are detectable for longer periods [14, 15], by poorer adherence to treatment in this age-group following the test and treat intervention [16–18] or by a persistently high force of infection [19] driven by the high HBR observed, as is suggested by the data (Fig. 2c). The increased rainfall recorded in 2019, which favoured the availability of breeding habitats, could explain part of the increase in malaria transmission levels observed in Riaba district, but not all.

**Fig. 8:**
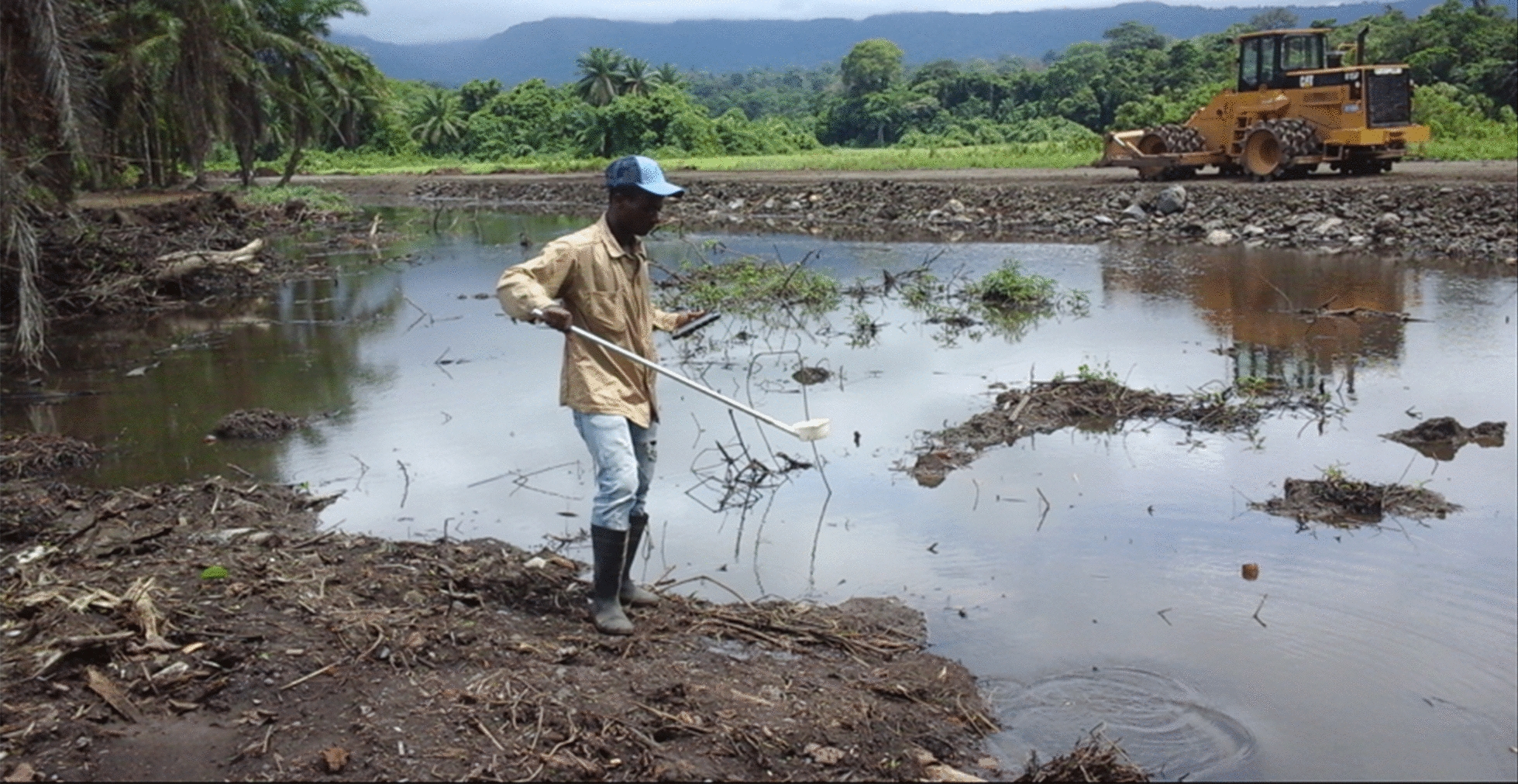
Construction site in Riaba district. Large water collections left unattended became active mosquito breeding habitats. In the picture, an entomologist is sampling the site for anopheline larvae. This was one of many such anthropogenic breeding habitats found in Riaba district in 2019

Notably, major road and real-estate development projects had been ongoing in Riaba for two to three months before the outbreak. Urban development and construction sites can potentially affect local vector ecology and thus require responsible management by sectors outside health care [20]. In fact, the main finding of the entomology teams during the intervention was the substantial number of anthropogenic mosquito breeding habitats created by these projects. These larval habitats were not only numerous but large and presented a significant challenge for LSM activities (Fig. 8). Although efforts were made to treat as many of them as possible, there were simply too many habitats spread over large areas, exceeding the manpower available to tackle them. Following the interventions, malaria prevalence decreased in most communities, but higher PR was seemingly clustered in those nearer to the construction sites (Figs. 6 and 7). A notable exception was Patio López, where PR measured during the MIS was higher than during the outbreak survey (35.7% *vs.* 3.3%), though only 14 people were sampled during the MIS in this community. Therefore, the confidence limits of this estimate were wide (95CI 12.8–64.9%) and true prevalence could well have been on the lower bound. On the other hand, the HBR estimated in Patio López, despite spiking at the beginning of the year alongside HBR estimated in most of the other entomological monitoring sentinel sites on the island, dropped by May 2019 and remained low thereafter. Conversely, the HBR in Patio Balboa remained high despite the interventions, which could be explained by the fact that this site is located in the surroundings of the anthropogenic breeding habitats created by construction projects. Though, historically, Patio Balboa has yielded particularly high vector densities, the current alterations to the local ecology may explain the persistently high HBR observed in this location.

Other, more pernicious drivers may have aggravated the outbreak in Riaba district. First, human mobility and parasite importation from mainland Equatorial Guinea, where malaria transmission is significantly more intense [21], have been identified as important contributors to the parasite prevalence observed on Bioko Island [22–24]. Apart from the generation of vector breeding habitats, the construction sites in Riaba demanded a high influx of migrant workers from mainland who could have been malaria infected and, therefore, could have increased the local parasite pool. Also, the presence of military camps in Riaba with highly rotating personnel determines a constant flux of people to and from mainland, potentially resulting in higher parasite importation. Second, the limited uptake of interventions by the population despite universal coverage may have also contributed to the problem. Data from annual MIS indicate that LLIN ownership and access constantly decrease from year to year, a problem that is ubiquitous across Bioko Island [1]. The last mass distribution campaign on Bioko took place in 2018, 6 months before the outbreak. During that campaign, LLINs were distributed to virtually all households on the island [25]. LLIN population access (*i.e.* availability of at least one LLIN for every two people) in Riaba, however, was estimated at 75.2% 2 months after distribution and only at 53.5% in 2019. Moreover, LLIN use, regardless of access, remains sub-optimal with 49.3% and 48.3% of the population surveyed in Riaba in 2018 and 2019 reporting to have slept under a LLIN the night before, decreasing the effectiveness of this vector control intervention [26]. Finally, changes in host-seeking behaviour of anopheline vectors as a response to indoor vector control interventions have been observed on the island [27]. In light of increased vector densities, outdoor biting could have amplified transmission.

In 2019, the malaria control strategy of the NMCP/BIMEP was redefined towards the goal of malaria elimination. Given the local vector ecology, however, malaria receptivity on the island remains high [22]. In 2019, EIR in Patio Balboa was more than ten-fold higher than in 2018 and, given that sporozoite rates did not increase in the same magnitude, this was mostly attributed to significant increases in vector densities. This suggests that, despite the great reductions in transmission intensity across Bioko in general, and in Riaba district in particular, malaria receptivity in these areas is indeed important. The outbreak in Riaba exposed several vulnerabilities that probably combined to produce the observed surge in malaria clinical cases. It showed that, if ecological conditions were to change or if interventions were relaxed, there will be a very high risk of malaria resurgence. Changing ecological conditions were revealed by the increase in HBR following rainfall anomalies and the increased availability of vector breeding habitats driven by the presence of large construction projects. Relaxing of interventions resulted from logistical constraints at the beginning of 2019 that delayed the start of malaria control activities, consequently weakening the monitoring and response capacities of the NMCP/BIMEP. The need of sustained malaria interventions with an emphasis on vector control cannot be underscored enough in this context, as cannot be the importance of multi-sector participation as an essential component of integrated strategies when it comes to the fight against malaria. This case study signals the heavy challenges ahead in the difficult path to malaria elimination on Bioko Island.

## Data Availability

The datasets used and analysed during the current study are available from the corresponding author on reasonable request.

## Abbreviations

BIMEP: Bioko Island Malaria Elimination Project
NMCP: National Malaria Control Programme
MIS: Malaria indicator survey
IRS: Indoor residual spraying
LLIN: Long-lasting insecticidal net
PR: Parasite rate
HBR: Human biting rate
EIR: Entomological inoculation rate
RDT: Rapid diagnostic test
IQR: Inter-quartile range
95CI: 95% confidence intervals
